# Differential Responses to Bioink-Induced Oxidative Stress in Endothelial Cells and Fibroblasts

**DOI:** 10.3390/ijms22052358

**Published:** 2021-02-26

**Authors:** Hatice Genç, Jonas Hazur, Emine Karakaya, Barbara Dietel, Faina Bider, Jürgen Groll, Christoph Alexiou, Aldo R. Boccaccini, Rainer Detsch, Iwona Cicha

**Affiliations:** 1 Section of Experimental Oncology and Nanomedicine (SEON), Else Kröner-Fresenius-Stiftung-Endowed Professorship for Nanomedicine, Department of Otorhinolaryngology, Head and Neck Surgery, University Hospital Erlangen, Friedrich-Alexander-Universität Erlangen-Nürnberg, 91054 Erlangen, Germany; Hatice.Genc@uk-erlangen.de (H.G.); c.alexiou@web.de (C.A.); 2 Institute of Biomaterials, Friedrich-Alexander-Universität Erlangen-Nürnberg, 91058 Erlangen, Germany; jonas.hazur@fau.de (J.H.); Emine.Karakaya@fau.de (E.K.); Faina.Bider@fau.de (F.B.); 3 Department of Cardiology and Angiology, University Hospital Erlangen, 91054 Erlangen, Germany; Barbara.Dietel@uk-erlangen.de; 4 Department of Functional Materials in Medicine and Dentistry, University Hospital Würzburg, 97070 Würzburg, Germany; juergen.groll@fmz.uni-wuerzburg.de

**Keywords:** alginate di-aldehyde, gelatin, oxidative stress, glutathione, cell viability, cell death

## Abstract

A hydrogel system based on oxidized alginate covalently crosslinked with gelatin (ADA-GEL) has been utilized for different biofabrication approaches to design constructs, in which cell growth, proliferation and migration have been observed. However, cell–bioink interactions are not completely understood and the potential effects of free aldehyde groups on the living cells have not been investigated. In this study, alginate, ADA and ADA-GEL were characterized via FTIR and NMR, and their effect on cell viability was investigated. In the tested cell lines, there was a concentration-dependent effect of oxidation degree on cell viability, with the strongest cytotoxicity observed after 72 h of culture. Subsequently, primary human cells, namely fibroblasts and endothelial cells (ECs) were grown in ADA and ADA-GEL hydrogels to investigate the molecular effects of oxidized material. In ADA, an extremely strong ROS generation resulting in a rapid depletion of cellular thiols was observed in ECs, leading to rapid necrotic cell death. In contrast, less pronounced cytotoxic effects of ADA were noted on human fibroblasts. Human fibroblasts had higher cellular thiol content than primary ECs and entered apoptosis under strong oxidative stress. The presence of gelatin in the hydrogel improved the primary cell survival, likely by reducing the oxidative stress via binding to the CHO groups. Consequently, ADA-GEL was better tolerated than ADA alone. Fibroblasts were able to survive the oxidative stress in ADA-GEL and re-entered the proliferative phase. To the best of our knowledge, this is the first report that shows in detail the relationship between oxidative stress-induced intracellular processes and alginate di-aldehyde-based bioinks.

## 1. Introduction

Biofabrication offers a great opportunity to fabricate tissue models with suitable complexity for regenerative medicine, cancer research, drug screening or disease modelling. This young research field encounters several challenges, one of which is related to the composition and properties of the bioinks that must serve as extracellular matrices (ECM) for cell organization [1]. Bioink development is challenging since an ideal ink formulation, usually based on hydrogel systems, needs to cover multifactorial biological, biomechanical and physicochemical properties. On one hand, bioinks must have the appropriate viscosity, stiffness and shape fidelity for the printing process [2,3]. On the other hand, they must be biocompatible, biodegradable and soft enough to mimic the natural ECM [4,5]. This complexity of requirements comes with the necessity of a comprehensive evaluation of hydrogels intended for bioink applications, both from the materials and cell biology sides. 

Owing to their structural similarity to the ECM and their hydrated structure that allows the required nutrient and gas exchange, different hydrogels are commonly favored for 3D biofabrication. Within a large family of natural hydrogels, alginate-based hydrogel systems have been extensively investigated due to their several advantages, such as good biocompatibility and mechanical properties, as well as cell-friendly crosslinking mechanism [6,7,8,9]. However, alginate alone is not suitable for 3D printing precise geometries and suffers from very poor cell-material interaction and tissue remodeling capability [10,11]. One strategy to overcome these limitations is a modification of the pristine alginate to obtain its oxidized product alginate di-aldehyde (ADA) [12], which is then subsequently crosslinked with gelatin (GEL) in order to improve cell adhesiveness and to control the biodegradability, hydrolytic properties, as well as shape fidelity of 3D printed structures, which can be achieved by changing the content of ADA and GEL and the extent of alginate oxidation [10]. This strategy of dynamic chemical crosslinking by Schiff base chemistry represents one of the current trends reported in the literature and has also been used with other natural materials [13,14]. ADA with different GEL contents has been reported to have advantages in several biomedical applications such as wound dressing [15], improvement of cartilage tissue formation [16], development of cell adhesive surfaces [17] and as injectable cell delivery vehicle for adipose tissue engineering [18]. However, although several previous studies showed good cytocompatibility of ADA-GEL with several cell types in 2D [19,20,21,22] and 3D [23,24,25,26,27], cytotoxic effects on endothelial cells were also observed [28]. In some reports [29,30], the cells remained in the growth arrest phase, or their viability decreased for up to several days before regaining the proliferative stage in 3D. It is unclear, however, whether the observed reduction in cell viability results from the process of alginate modification, whether the remnants of toxic reagents used in the oxidation procedure are insufficiently removed or whether it results from the presence of aldehyde groups created during the modification of the natural material. 

In this study, we hypothesized that free aldehyde groups in the hydrogel could be responsible for reduced cell viability and can elicit a cell type-specific reaction. Exogenous aldehydes can stress cells and lead to DNA damage through oxidative stress [31,32]. At the cellular level, this oxidative stress can result in a wide range of responses such as inhibition of proliferation, growth arrest or even cell death. Cells contain a number of antioxidant enzymes (e.g., superoxide dismutase, catalase and glutathione peroxidase) and nonenzymatic antioxidants (e.g., glutathione, retinoic acids, vitamins C and E) to minimize or reverse the effect of oxidative stress. However, when the amount of reactive oxygen species (ROS) exceeds the antioxidant capacity of cells, cell damage is no longer reversible. The severity of this damage highly depends on the amount of ROS and the cell type [33]. Intermediate doses of ROS can cause a temporary or permanent cell growth arrest. On the other hand, high doses of ROS result in cell death via either apoptotic or necrotic mechanisms [34]. Therefore, survival and proliferation of cells in any kind of environment are highly dependent on the balance between exogenous oxidative stress present in the environment and the antioxidant capacity of the cell itself. To evaluate our hypothesis, we used several different cell types, including immortalized and primary fibroblasts and endothelial cells. In the preliminary experiments, two cell lines commonly used for biocompatibility studies were grown in alginate or ADA with different degrees of oxidation to evaluate whether the content of aldehyde groups affects their viability. Subsequently, primary human cells were cultured in ADA-based hydrogels to investigate the molecular effects of the oxidized material in conditions closer to physiological situation and to determine the cell type-specific response to oxidative stress. To the best of our knowledge, this is the first report that focuses on the oxidative stress-mediated intracellular reactions induced in alginate di-aldehyde-based hydrogels. 

## 2. Results

### 2.1. Hydrogel Characterization

**Figure 1:**
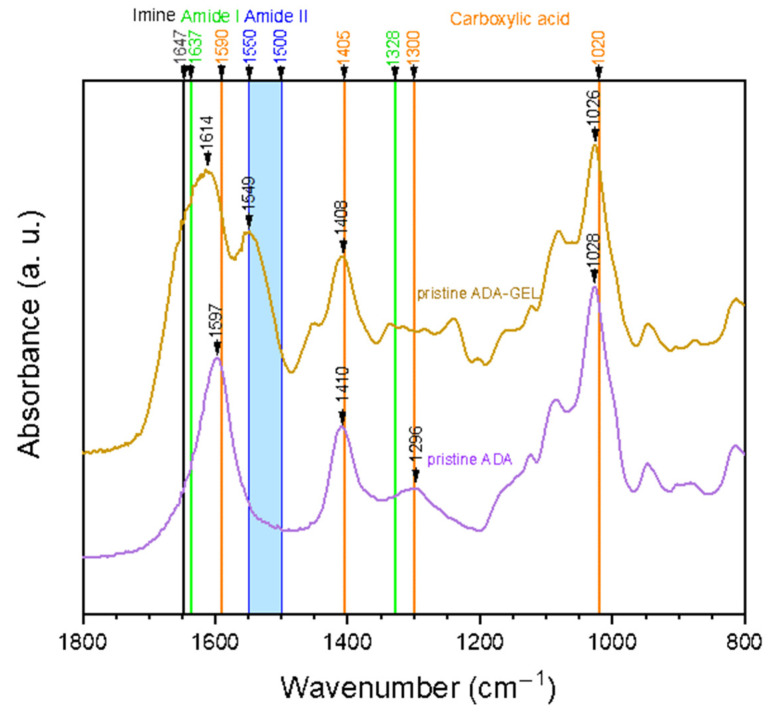
ATR-FTIR spectra of pristine alginate di-aldehyde (ADA) and alginate covalently crosslinked with gelatin (ADA-GEL). The relevant peaks are discussed in the text.

Figure 1 shows FTIR spectra of pristine ADA and ADA-GEL hydrogels. It can be noted that carboxylic acid peaks are dominant in the pure ADA sample. Its peaks indicating C=O asymmetric bonds (1590 cm^−1^), as well as symmetric (1405 cm^−1^) stretching vibration are clearly visible. Additionally, peaks at 1300 cm^−1^ and 1020 cm^−1^, which are known to relate to the C-O stretching vibration of the carboxyl groups, are present in the spectrum at 1296 cm^−1^ and 1028 cm^−1^, respectively [35]. In comparison to pure ADA, additional peaks can be found in the ADA-GEL spectrum. On one hand, a peak at 1549 cm^−1^ emerges, which falls into the region of amide II band (1500–1550 cm^−1^) [36]. Considering these results, the presence of gelatin amide groups is clearly verified.

On the other hand, the main peak of ADA at 1597 cm^−1^ shifts to 1614 cm^−1^ and becomes broader. As the C=O stretching of amide I (1637 cm^−1^), as well as the C=N stretching vibration of imine (1623–1647 cm^−1^) are expected in the same region, the peak shift and broadening is most likely attributed to an overlay of peaks. The C=N stretching vibration, which indicates the successful formation of a Schiff base (imine) bond, has been shown to occur in the range of 1623–1647 cm^−1^ in various tissue engineering applications [10,37,38,39,40]. Specifically, Sarker et al. published similar spectra before, suggesting the formation of Schiff base in their ADA-GEL hydrogels [10]. Moreover, a study by Yuan et al. stated that the occurrence of a peak at 1647 cm^−1^ indicated the successful formation of imine bonds in ADA-amino gelatin [37].

**Figure 2:**
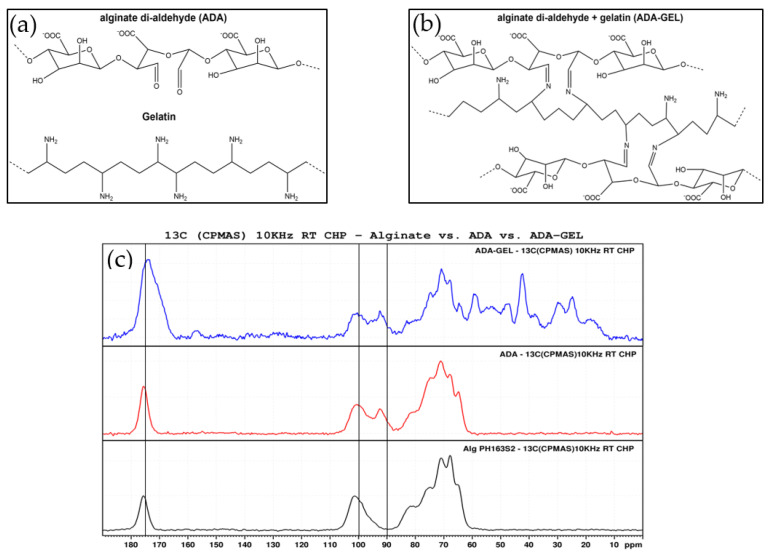
Schematic depiction of (**a**) ADA and gelatin separately and (**b**) ADA-GEL structure formed via Schiff base bond formation. (**c**) Solid state 13C NMR spectra of ADA-GEL, pristine ADA and alginate (from top to bottom).

In Figure 2a,b, schematic depictions of the molecular structures of ADA, gelatin and ADA-GEL are shown. Solid state 13C NMR spectra of pristine samples of alginate, ADA and ADA-GEL can be observed in Figure 2c. The peaks in the region of 60–80 ppm can be assigned to the pyranose carbons of the alginate backbone and are found in all samples. Also present in all spectra are the peaks at 100 and 175 ppm, representing hydroxyl and carboxyl carbon, respectively. In the oxidized state of alginate, a new peak at 93 ppm appears in the ADA and ADA-GEL samples. This peak can be assigned to the hemiacetal carbon. It is known that the hemiacetal group is in imbalance with free aldehyde groups, with the cyclic hemiacetal state being strongly favored [12,41]. The equilibrium between the aldehyde and hemiacetal state of ADA is also largely dependent on the moisture content of the samples, with a moist environment favoring the aldehyde state [41]. Thus, a successful oxidation of alginate is indicated by these peaks, as samples were measured in dry state. Further peaks of ADA-GEL in the region of 10–60 ppm can all be assigned to the presence of gelatin.

### 2.2. Evaluation of Cell Viability as a Function of Oxidation Degree

**Figure 3:**
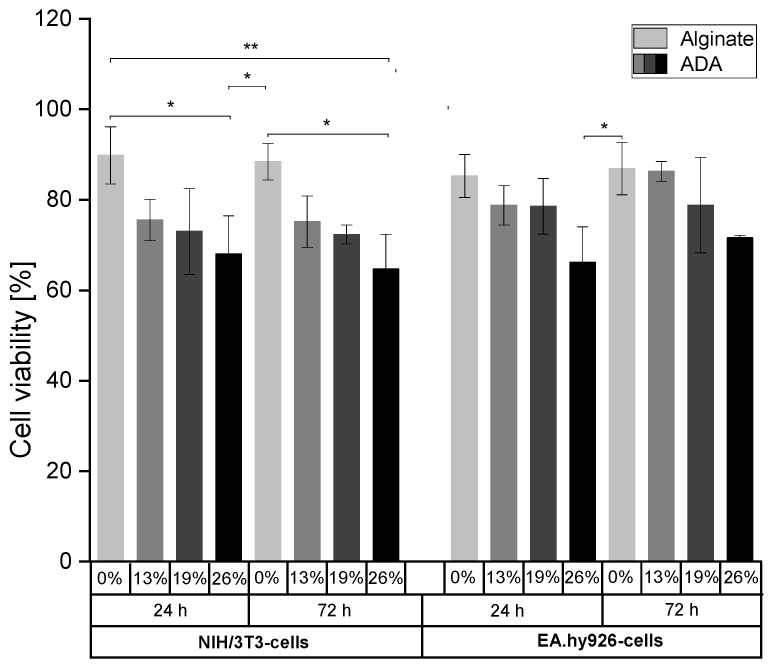
Cell viability of NIH/3T3- (left) and EA.hy926-cells (right) embedded in alginate and ADA hydrogels (%DO: 13–26%) after incubation for 24 h and 72 h. * *p* < 0.05, ** *p* < 0.01.

NIH/3T3-cells and EA.hy926-cells were grown for 24 h or 72 h in pure alginate (3% (w/v)) or ADA hydrogels (5%(w/v)) with different degrees of oxidation (%DO) in order to evaluate whether the content of aldehyde groups affects cell viability. In this regard, different %DO (0–26%) were used to investigate the interactions between CHO groups and both cell lines by determining the cell viability using Calcein AM and DAPI staining (Figures S1 and S2). Figure 3 revealed an association between the content of CHO groups and cell surviving for both time points and in both cell lines. The highest cell viability rates were observed in alginate hydrogels (>80%). With an increase in %DO from 13% to 26%, the number of viable cells decreased and reached a minimum value in the most oxidized ADA hydrogels (>55% viability). 

### 2.3. Mechanisms of Oxidized Material-Induced Cytotoxicity in Primary Human Cells

Subsequently, we used primary human cells grown in pure ADA and ADA-GEL hydrogels at 13% DO to investigate the molecular effects of oxidized material and to determine the cell type-specific response to oxidative stress. In order to perform the flow cytometric analyses required for the determination of cell viability and the mechanisms of cell death, it was necessary to isolate the embedded cells from the hydrogels, which precluded the use of Alg as control in these experiments. Human fibroblasts and primary ECs were grown in ADA (2.5 % (w/v)) and ADA-GEL (2.5–2.5% (w/v)) hydrogels for 6 h, 24 h and 72 h. These concentrations were based on our previously published experimental set-up with primary human cells [42]. 

**Figure 4:**
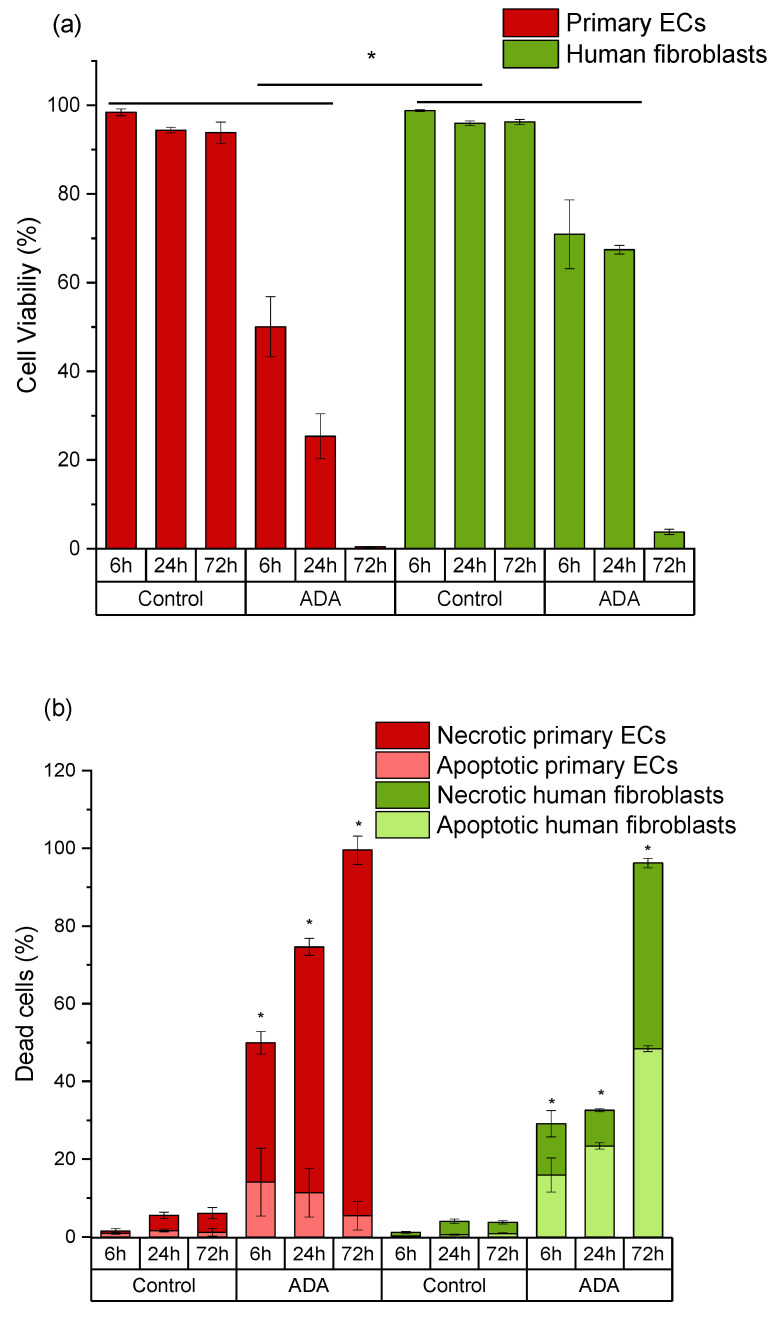
Time-dependent comparison of primary endothelial cells (ECs) and fibroblasts grown in ADA. (**a**) Cell viability (DiI-positive cells); (**b**) apoptotic populations in total death cluster (DiI-negative, PI-negative staining) and necrotic populations in total death cluster (DiI-negative, PI-positive staining). Control: cells with DCFH-DA grown on plastic. * *p* < 0.05 indicates significant differences between the groups in cell viability (**a**) and number of apoptotic cells (**b**).

In ADA hydrogels, cell viability was relatively unchanged for fibroblasts after 6 h and 24 h of incubation, but the numbers of viable cells dramatically decreased after 72 h (Figure 4a). Cell death of fibroblasts grown in ADA mainly occurred via apoptosis, and necrotic cell number increased upon extended time of incubation (Figure 4b). On the other hand, the viability of primary ECs decreased instantly within 6 h in pure ADA hydrogel and all cell populations died within 72 h (Figure 4a). The main mechanism of cell death was determined to be necrosis (Figure 4b). It must also be noted, that the cytotoxic effects of ADA were overall much stronger in primary cells than those observed in cell lines. After 72 h of incubation, about 55% of cell line cells were still viable. In contrast, the viability of primary ECs was reduced to 0% and the viability of primary fibroblasts was below 10% at the same time point.

**Figure 5:**
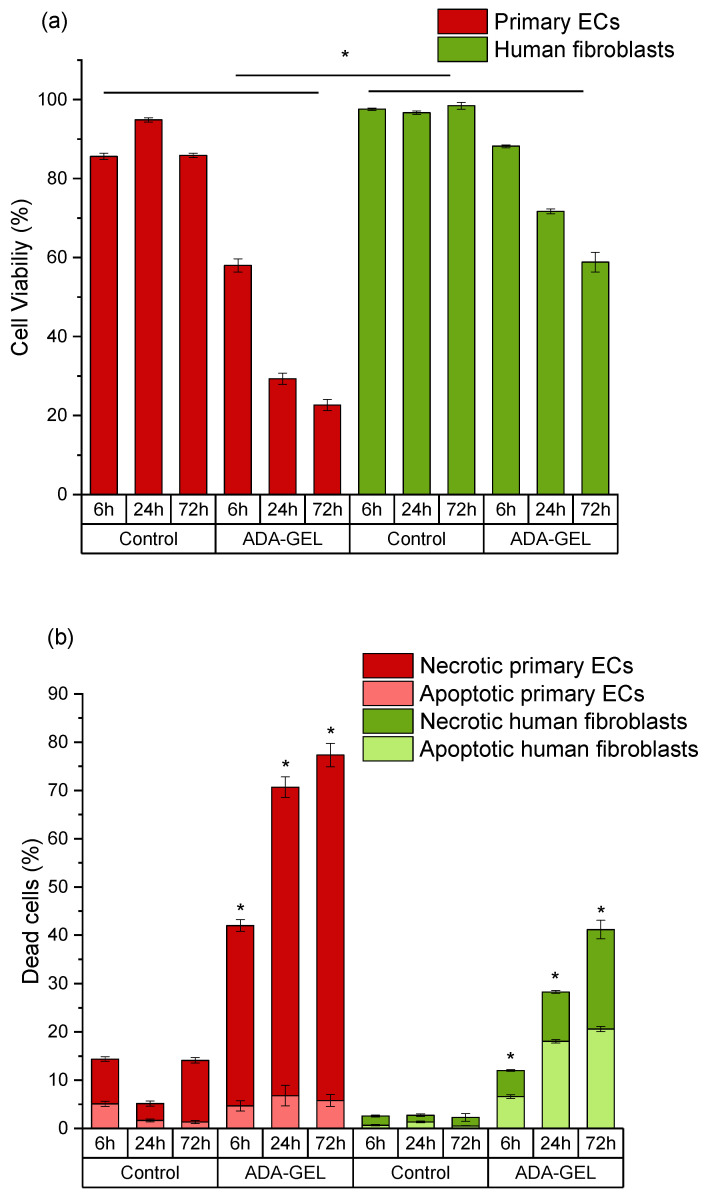
Time-dependent comparison of primary ECs and fibroblasts grown in ADA-GEL. (**a**) Cell viability (DiI-positive cells); (**b**) apoptotic populations in total death cluster (DiI-negative, PI-negative staining) and necrotic populations in total death cluster (DiI-negative, PI-positive staining). Control: cells with DCFH-DA grown on plastic. * *p* < 0.05 indicates significant differences between the groups in cell viability (**a**) and number of necrotic cells (**b**).

Fibroblasts showed 88% of cell viability after 6 h of incubation in ADA-GEL, and cell viability decreased in a time-dependent manner; however, unlike in ADA systems, viability never went below 50%, even after 72 h of incubation (Figure 5a). In fibroblasts, cell death mainly occurred via apoptosis at 6 h and 24 h. However, extended time of incubation increased the number of necrotic cells similar to that in the ADA systems (Figure 5b). On the other hand, primary ECs grown in ADA-GEL showed reduced cell viability after 6 h of incubation (58%) and the numbers of viable cells decreased dramatically in a time-dependent manner (Figure 5a). In contrast to fibroblasts, cell death of primary ECs mainly occurred via necrosis (Figure 5b).

**Figure 6:**
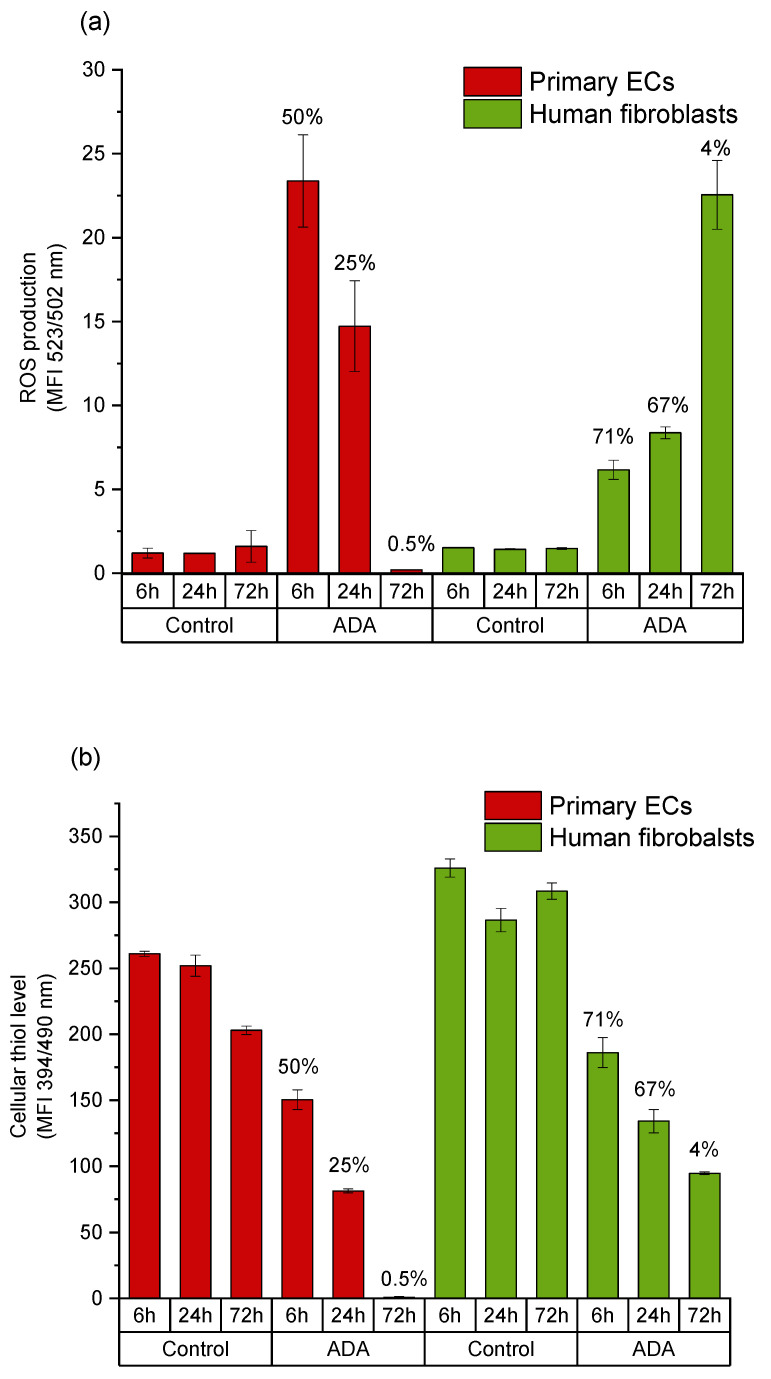
Comparison of intracellular reactive oxygen species (ROS) levels and cellular thiol levels in primary ECs and fibroblasts grown in a time dependent manner in ADA. (**a**) Mean value of DCF emission detected by flow cytometer; (**b**) intracellular thiol level of cells under the same conditions. Results obtained in the same cell population with MBB staining and mean value of emission are presented. Cell viability at respective time points is indicated above the columns. Control: cells with DCFH-DA grown on cell culture plastic.

**Figure 7:**
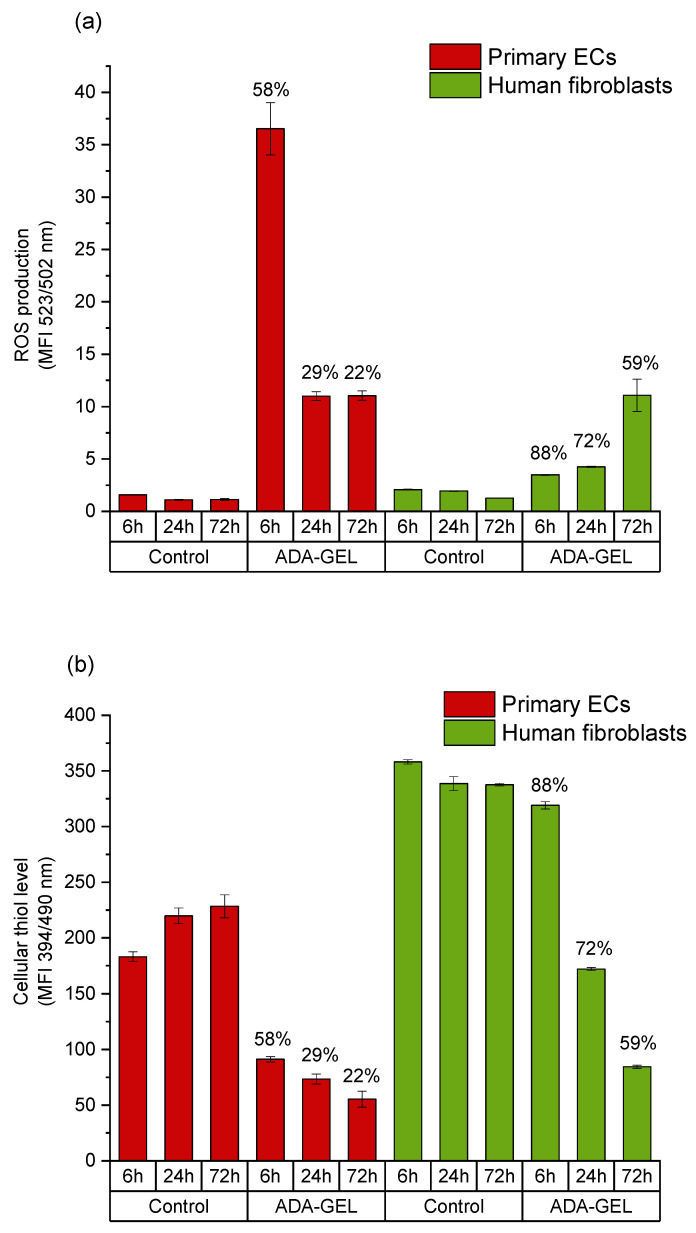
Comparison of intracellular ROS levels and cellular thiol levels in primary ECs and fibroblasts grown in a time dependent manner in ADA-GEL. (**a**) Mean value of DCF emission detected by flow cytometer; (**b**) intracellular thiol content of cells under the same conditions. Results obtained in the same cell population with MBB staining and mean value of emission are presented. Cell viability at respective time points is indicated over the columns. Control: cells with DCFH-DA grown on cell culture plastic.

Intracellular ROS generation was determined via de-esterification reaction of DCHF-DA to DCF upon oxidation in viable cell population. After 6 h incubation, the generation of ROS in fibroblasts grown in ADA-GEL was only slightly higher than in control cells. On the other hand, primary ECs showed an amount more than seven times higher of ROS generation in ADA-GEL and about three times higher ROS generation in ADA in comparison to fibroblasts after 6 h of incubation. However, in both cell types, the amount of intracellular ROS remained higher than in the control groups independent of incubation time (Figure 6 and Figure 7). GSH activity of viable fibroblasts in ADA-GEL was nearly three times higher at 6 h and two times higher at 24 h as compared with that of ECs. Results also showed that control human fibroblasts had much higher cellular thiol level than primary ECs (Figure 6 and Figure 7). This finding may be related to the poor survival of ECs in both ADA and ADA-GEL hydrogels.

**Figure 8:**
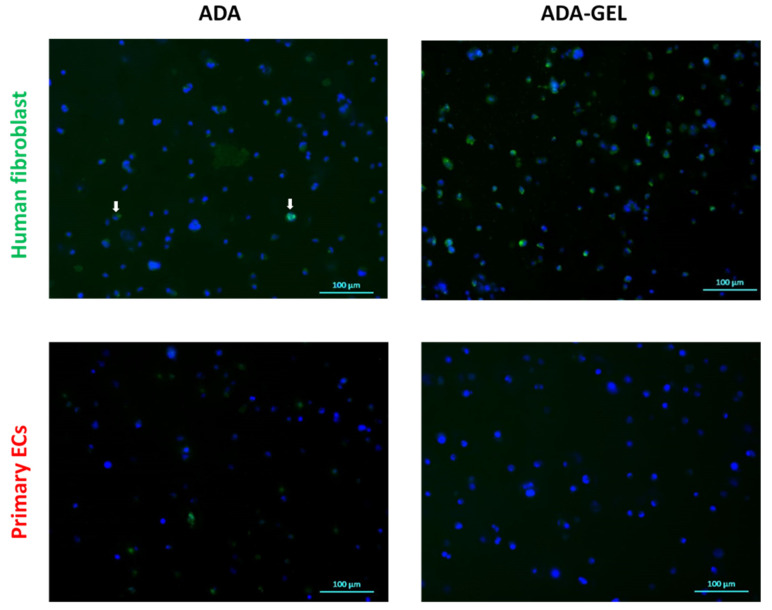
Immunofluorescence staining of proliferating cells (green channel) after 72 h of incubation. Representative images show Ki-67 expression in primary ECs and human fibroblast grown in ADA and ADA-GEL. Nuclei: Hoechst (Blue), Ki-67: Alexa Fluor 488 (green). The white arrows indicate scarce fibroblasts in ADA positive for Ki-67.

Proliferation potential of cells was assessed by analyzing Ki-67 protein expression in primary ECs and fibroblasts grown in the ADA and ADA-GEL system for 6 h, 24 h and 72 h. The expression of Ki-67 was determined by immunocytochemical staining. No significant protein expression was detected at 6 h and 24 h of incubation for both cell types and hydrogel systems. At 72 h, a small number of Ki-67 positive fibroblasts were detected in ADA hydrogels (Figure 8, upper left image), whereas many proliferating fibroblasts (positive for Ki-67) were observed in ADA-GEL hydrogels (Figure 8, upper right image). In contrast to fibroblasts, no proliferating primary ECs were detectable in either ADA or ADA-GEL after 72 h of incubation.

### 2.4. ATR-FTIR of Cell-Containing Hydrogels

**Figure 9:**
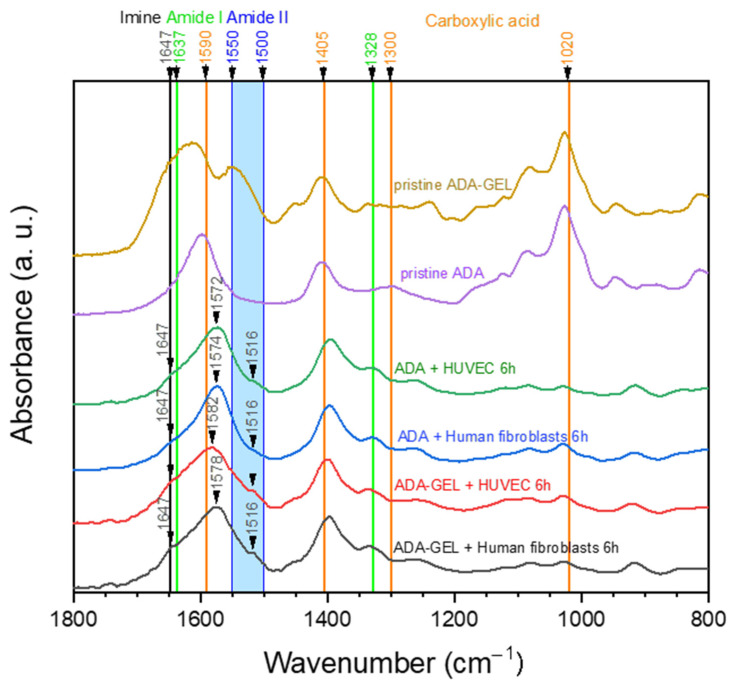
FTIR spectra of hydrogel samples from cell culture experiments after 6 h of incubation in comparison to pristine ADA and ADA-GEL. Cells were removed from the hydrogels prior to measurements, and “+ HUVEC” indicates samples that were obtained from cell culture with primary ECs. The characteristic peaks are discussed in the text.

Figure 9 shows FTIR spectra of hydrogel samples that were separated from cells after 6 h of incubation in comparison to pristine ADA and ADA-GEL. Due to the fact that 24 h and 72 h samples showed similar characteristics to the 6 h samples, they were excluded to simplify the graph. All samples obtained from cell culture seem to show similar characteristics at first sight (Figure 9). In comparison to pristine ADA, new peaks or shoulders of the dominant C=O asymmetric peak of carboxylic acid can be found in the region of N-H stretching vibration of amide II (1500–1550 cm^−1^) [36]. This points to the presence of amide II in all cell culture samples, thus indicating the presence of gelatin or other proteins. As the samples “ADA + Human fibroblasts” and “ADA + HUVECs” did not contain any proteins in the first place, the FTIR spectra indicate that serum proteins which are suspended in the cell culture medium are present inside the hydrogel during culturing. In addition, a small shoulder at 1647 cm^−1^ can be observed, which can be attributed to Schiff base formation [37]. However, one can observe that absorptions in the region of the amide II and imine peaks are much lower when comparing the spectra of the cultured samples to those of pristine ADA-GEL. This leads to the assumption that considerable amounts of gelatin used for ADA-GEL preparation have already been released after 6 h of incubation. It is assumed that this gelatin release refers to the amount of excessive gelatin, which is not cross-linked via a Schiff base bond. On closer examination, a slight tendency to more distinct amide II (1516 cm^−1^) and imine (1647 cm^−1^) peaks in ADA-GEL samples can be observed in comparison to ADA samples.

Thus, our hypothesis is that most of the gelatin in ADA-GEL is not bound to aldehyde groups and hence released in a short time, while a small amount is bound in the hydrogel via imine bonds, which occupy free aldehyde groups. This has been also suggested in previous studies on ADA-GEL hydrogels [12].

## 3. Discussion

The survival of cells during the biofabrication process depends on several factors, among them are the shear stress during extrusion printing and the mechanical stress acting on the cells in subsequent 3D culture, but also on the bioink chemistry and composition itself. ADA is a commonly used bioprinting material owing to its hydrogel-forming properties, suitable shape-fidelity and overall good biocompatibility [11,18,19,22,26,43,44]. However, in our previous studies cell type-dependent toxicity of ADA-based hydrogels was observed [42], although the molecular mechanisms of this effect were unknown. We hypothesized that the cytotoxic effect of ADA may result from an increased oxidative stress induced by the presence of aldehyde groups. To clarify this assumption, we first grew two immortalized cell types originating from mouse fibroblasts (NIH/3T3) and human immortalized vascular cells (EA.hy926) in ADA hydrogels with an increasing degree of oxidation and observed their influence on cell viability. The chosen cell lines are among the most commonly used types of immortalized fibroblasts and endothelial cells, having the advantage of the possibility of high passaging and the relative ease of culture. As the research in the field of material science utilizes these cell lines, e.g., for evaluation of material biocompatibility, they are broadly available and thus constitute a good point of reference for other researchers. In both NIH/3T3 and EA.hy926 cells, there was a concentration-dependent effect of oxidation degree on cell viability, with the strongest cytotoxicity observed at the highest DO of 26% after 72 h of culture. It is known from the literature that CHO-groups may be responsible for cytotoxicity [31,45]. Besides certain alterations in material properties, e.g., viscosity and chain length, the main difference between alginate and ADA hydrogels resulting from the treatment with NaIO_4_ was the formation of CHO groups. Together with the extremely stringent purification steps to endure the removal of the toxic oxidation reagents, this indicates that it was the presence and amount of aldehyde groups that directly affected cell viability.

Interestingly, there were also differences between the NIH/3T3 cell line derived from mouse fibroblasts and the immortalized EA.hy926 human vascular cells, whereby the latter were less sensitive to low-oxidized ADA (13% DO). This effect was rather unexpected, as our previously reported data have shown a strong cytotoxic response to ADA-based inks in primary human ECs but not in human fibroblasts [42]. Therefore, in order to clarify the mechanisms of ADA-induced responses in primary human cells, more detailed investigations including the viability, cell death mechanisms, ROS generation and cellular thiol status were performed in primary ECs and fibroblasts. A further reason for using primary cells was related to the fact that the tested biomaterials are intended for potential application in humans. This warrants evaluation of their interactions with primary human cells, which possess morphological and functional characteristics of their native tissue. 

As shown in Figure 4 and Figure 5, the cytotoxic effect of ADA (13% DO) on human ECs was confirmed in the present study. An extremely strong ROS generation resulting in a rapid depletion of cellular thiols was observed already after 6 h of incubation, which reduced the amount of viable cells by 50 %. In contrast, less pronounced effects of ADA were noted in human fibroblasts, whereby the ROS generation at 6 h was lower than in ECs, and the viability was reduced by 30%. This result can be related to the higher cellular thiol content of fibroblasts, which has been previously reported by Lorenz et al. [46]. and was also confirmed in our study. Compared to primary ECs, fibroblasts consistently showed a much higher baseline thiol level in all measurements performed with comparable numbers of control cells. High antioxidant capacity of primary human fibroblasts was previously reported in relation to their resistance against mild oxidative stress and explains how these cells could escape the growth arrest by extending the replicative life span [47]. A major difference between ECs and fibroblasts was also observed in terms of correlation between ROS production and the cellular thiol levels. The correlation observed in fibroblasts reflects the natural response of healthy and robust cells to oxidative stress, whereby gradually increasing ROS production leads to the depletion of thiols, in order to limit or prevent the oxidative damage. A different effect was observed in ECs grown in ADA, as the massive oxidative assault leads to survival of only 50% of cells within merely 6 h. In these conditions, only the cells which still have relatively high thiol content can remain viable despite very high ROS levels. Similar was seen after 24 h, whereby only 25% ECs remained viable, which was presumably the most robust part of the total cell population, still containing the sufficient level of thiols. As nearly all ECs were dead at 72 h, only negligible levels of ROS or thiols were measurable at that time point.

Moreover, differential mechanisms of cell death induced by the oxidative stress were observed in the present study. Cell death of primary ECs mainly occurred via necrosis. A study of Simon and Fernández reported that ECs are highly sensitive to ROS and that the early stage of ROS production causes an extensive amount of necrotic cell death [48]. On the other hand, activation of an intracellular death program (apoptosis) was the main mechanism of cell death in the early stages of oxidative stress exposure (6 h and 24 h) in fibroblasts. In these cells, the percentage of necrotic cells was initially markedly lower than that of apoptotic cells, and increased gradually to match the apoptotic cell numbers after 72 h of incubation. These results can be explained by the oxidative stress-induced growth arrest of fibroblasts. Mammone et al. investigated this phenomenon in detail, showing that under oxidative stress conditions, fibroblasts go into non-proliferative state of senescence and cannot regain the replicative stage again [49]. Once this happens, fibroblasts secrete high amounts of apoptotic factors and enter the programmed cell death [49]. 

**Figure 10:**
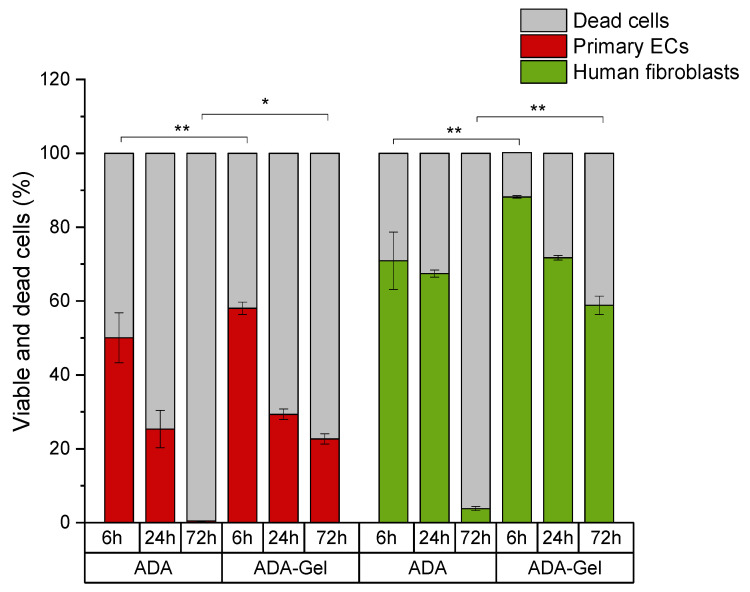
Comparison of cell viability between primary ECs and fibroblasts grown in ADA versus ADA-GEL. * *p* < 0.05, ** *p* < 0.001.

The presence of gelatin in the ADA hydrogel improved the primary cell survival, likely by reducing the oxidative stress via binding to the CHO groups. Consequently, ADA-GEL was better tolerated than ADA alone, in particular by human fibroblasts, the viability of which was maintained above 55% even after 72 h of incubation (Figure 10). The considerable cytotoxic effect was still noted in primary ECs, albeit to a lesser extent than in the case of pure ADA hydrogel. After 72 h of incubation, EC viability was maintained at the level of about 25%, as compared to 0% in ADA.

This increased survival of cells in ADA-GEL compared to pure ADA is likely due to the shielding of aldehyde groups by the primary amine groups in gelatin. As shown previously, gelatin can bind to ADA via Schiff base (imine bond) formation and thus free aldehydes which are known to decrease cell viability can be removed from the hydrogel system [10,37]. Nevertheless, distinct differences of FTIR spectra in Figure 1 indicate that parts of the gelatin content in the hydrogel system remain unbound to ADA and are thus removed from the hydrogel upon dissolution at 37 °C. Moreover, the formation of an imine bond is known to be reversible, depending on the environment of the system including pH, solvent and steric factors [50]. As the present hydrogel system is buffered to a pH of 7.4 by the cell culture medium, and the flexibility of amine groups is comparably low due to the cross-linked state of macromolecules, we hypothesize that few free aldehyde groups are present in the ADA-GEL hydrogel. Nevertheless, due to the reversible nature of the imine bond, a portion of free aldehyde groups remaining in ADA-GEL hydrogels may affect the cells, leading to an increased ROS production and oxidative challenge. Under these conditions, cells characterized by low antioxidant capacity (such as primary ECs) remain sensitive to ROS attack and respond with necrotic cell death. In contrast, cells with high antioxidative capacity, e.g., fibroblasts, are able to survive the oxidative stress and re-enter the proliferative phase. The analysis of Ki-67 protein expression confirmed that human fibroblasts regained proliferative state after 72 h of incubation in ADA-GEL system, as opposed to ECs. Although no previous reports exist to evaluate the kinetics of fibroblast proliferation upon exposure to oxidized materials, our results are in accordance with a recent study of Erdem et al., who showed that although fibroblasts viability was reduced by 72 h of hypoxic stress in a 3D gelatin methacryloyl system, the cells regained metabolic activity after 7 days [51]. 

## 4. Materials and Methods

### 4.1. Materials 

Sodium alginate, approved as a pharmaceutical excipient (Vivapharm PH163 S2), was obtained from JRS PHARMA GmbH & Co. KG, Germany. Sodium (meta) periodate (NaIO_4_ – BioUltra, ≥99.5%), ethanol (99.8%), ethylene glycol (Spectrophotometric grade, ≥99%), CaCl_2_ and gelatin (Type A, from porcine skin, Bloom 300) were purchased from Sigma-Aldrich Chemie GmbH (Munich, Germany). Dulbecco’s Phosphate Buffered Saline (DPBS; w/o Mg, w/o Ca) was from Gibco Life Technologies, Thermo Fisher (Schwerte, Germany). Dialysis tubes (Spectra/Por^®^1 Dialysis Membrane, MWCO: 6–8 kDa) were obtained from SpectrumLabs and the 0.45 µm and 0.22 µm Millipore filters (Rotilabo-syringe filters, PVDF) from Carl Roth (Karlsruhe, Germany). Ultrapure water (UPW, Direct-Q) was from Merck Millipore.

Endothelial cell growth medium and endothelial growth supplement were purchased from Promo Cell (Heidelberg, Germany). Dulbecco’s Modified Eagle’s Medium (DMEM) with high glucose and Trypsin–EDTA were purchased from PAN Biotech (Aidenbach, Germany). Accutase was obtained from Biowest (Nuaillé, France). Fetal bovine serum, phosphate buffer saline, Hank’s Balanced Salt Solution, penicillin, streptomycin, mouse anti-human Ki-67 monoclonal antibody, propidium iodide, 2′,7′-Dichlorofluorescin diacetate (DCFH-DA) and Triton™ X-100 were purchased from Sigma-Aldrich. MUSE^®^ Count &Viability Assay Kit was purchased from Merck-Millipore (Darmstadt, Germany). DiI (1,1′,3,3,3′,3′-hexamethylindodicarbocyanine iodide [DiIC1(5)]) was obtained from Life Technologies (Darmstadt, Germany). Monobromobimane (MBB, 3-(bromomethyl)-2,5,6-trimethyl-1H,7H-pyrazolo[1,2-a]pyrazole-1,7-dione), goat anti-mouse IgG (H+L) secondary antibody conjugated with Alexa Fluor Plus 488 were purchased from Thermo Fisher. Ringer’s solution was obtained from Baxter Healthcare (Zurich, Switzerland). Hoechst 33342, DAPI staining solution and Calcein AM were purchased from Invitrogen, Thermo Fisher.

### 4.2. Synthesis of Alginate Di-Aldehyde (ADA)

A partial oxidization of sodium alginate with sodium (meta) periodate (NaIO_4_) as oxidizing agent was performed in order to obtain alginate dialdehyde (ADA). In brief, 10 g of alginate were dispersed in 50 mL of ethanol (99.8 %) and kept under stirring in dark conditions. Then, appropriate amounts of NaIO_4_ (1.337, 2.003 or 2.674 g) were dissolved in 50 mL ultrapure water in the absence of light to achieve a corresponding oxidation degree of 13, 19 or 26%. The NaIO_4_ solution was added dropwise to the stirring alginate suspension immediately upon dissolution. After 6 h of oxidation time, the reaction was quenched by the addition of 10 mL ethylene glycol while further stirring at room temperature (RT) for an additional 30 min. Finally, the suspension was left 10 min for sedimentation of the ADA product and the supernatant was decanted. For the purification of ADA, it was dialyzed against ultrapure water in containers holding a volume of 15 L. The water was replaced with fresh water daily, and dialysis tubes had a molecular weight cut-off of 6–8 kDa. After 5 days, the purified ADA solution was frozen for 72 h at −20 °C and subsequently lyophilized (ALPHA 1−2 LDplus, CHRIST Gefriertrocknungsanlagen, Harz, Germany) to obtain a dry product.

### 4.3. Hydrogel Formulations

In order to prepare ADA 2.5 % (w/v) hydrogels, ADA dry product was dissolved in DPBS and stirred until completely dissolved, followed by filtration with a 0.45 µm Millipore filter. Similarly, ADA (5 % w/v) was synthesized for ADA-GEL preparation. Additionally, 5 % (w/v) gelatin was dissolved in DPBS at 37 °C and subsequently filtrated with a 0.22 µm Millipore filter. Afterwards, the gelatin solution was mixed with ADA 5 % (w/v) in a sterile beaker at a volume ratio of 1:1 for 10 min at 37 °C, to allow for Schiff base formation. Thus, the final concentration of ADA-GEL hydrogel was 2.5 % (w/v) ADA and 2.5 % (w/v) GEL.

### 4.4. Chemical Characterization of ADA and ADA-GEL

#### 4.4.1. Fourier-Transform Infrared Spectroscopy

An IRAffinity-1S Fourier transform infrared spectrometer (Shimadzu, Kyoto, Japan) provided with a quest-ATR unit and a diamond crystal was used to investigate the chemical composition of ADA-GEL and ADA samples. A total of 40 scans per spectrum at a resolution of 4 cm^−1^ were recorded in absorbance mode with Happ-Genzel apodization, to obtain attenuated total reflectance Fourier transform infrared (ATR-FTIR) intensity spectra. Prior to the measurements, all samples were frozen at −20 °C for at least 24 h and afterwards lyophilized for 24 h using a freeze-dryer (ALPHA 1−2 LDplus, CHRIST Gefriertrocknungsanlagen).

#### 4.4.2. Solid State 13 C NMR Spectroscopy

To confirm the formation of aldehyde groups within the ADA chains, solid state 13C NMR spectra were acquired using Bruker Advanced spectrometer (Bruker Biospin GmbH, Germany) [52]. For this purpose, the zirconia rotors were packed with 100 mg solid products and sealed with caps. All samples were spun at 10 Hz and Larmor frequencies of 100 MHz for 13C nuclei were operated. Using TopSpin software, all signals in the ADA spectra were analyzed, where the x axes represent the chemical shift and y show the signal intensity.

### 4.5. Cell Culture

#### 4.5.1. Endothelial Cells

Human endothelial cell lines (EA.hy926 cells) obtained from ATCC were cultured in DMEM supplemented with 10% (v/v) FCS and 1% (v/v) penicillin streptomycin and 1% (v/v) L-glutamine at 37 °C with a controlled atmosphere of 5% CO_2_ and 95% relative humidity.

Primary human umbilical vein endothelial cells (ECs) were isolated from freshly collected umbilical cords by a standard technique [53]. Isolated cells were maintained in endothelial cell growth medium (Promo Cell) with endothelial cell growth supplement containing 5 % fetal calf serum, 4 µL/mL heparin, 10 ng/mL epidermal growth factor and 1 µg/mL hydrocortisone in a humidified 5 % CO_2_ incubator. The use of human material was approved by the Ethics Committee of the Faculty of Medicine at the University of Erlangen-Nürnberg (case no. 246-13B). All subjects enrolled in this research have given an informed consent according to the ethical guidelines. In all experiments, ECs at passage 1–2 were used.

#### 4.5.2. Fibroblasts

Commercially available mouse embryonic fibroblast cells (NIH/3T3) were cultured in high glucose DMEM supplemented with 10% (v/v) bovine calf serum (BCS) and 100 U/mL penicillin streptomycin, 4 mM L-glutamine and 1 mM sodium pyruvate at 37 °C with a controlled atmosphere of 5% CO_2_ and 95% relative humidity.

Commercially available normal human dermal fibroblasts (PromoCell) were cultured in DMEM supplemented with 10 % (v/v) FCS and 1 % (v/v) penicillin streptomycin, at 37 °C with a controlled atmosphere of 5 % CO_2_ and 95 % relative humidity.

### 4.6. Cell Seeding in the Hydrogels

In order to investigate the influence of aldehyde (CHO) groups on cell vitality, NIH/3T3 and EA.hy926 cells (1 × 10^6^/mL) were embedded in alginate and ADA-solutions of different oxidation degrees (%DO, 13–26%). Using a high viscous pipette, 200 μL of all bioinks were transferred into cell culture well-plates and were crosslinked with CaCl_2_ (0.1 M) for 10 min and incubated at 37 °C with a controlled atmosphere of 5 % CO_2_ and 95% relative humidity.

For detailed investigations of molecular mechanisms of cell response to oxidized hydrogels, human fibroblasts and primary ECs were harvested and counted in Muse^®^ Cell Analyzer using MUSE^®^ Count & Viability Assay Kit. Subsequently, cell suspensions containing 1 × 10^6^ cells were mixed with 1 mL of the appropriate hydrogel (ADA-GEL or ADA, both at 13% DO) and casted in 24 well plates with a final volume of 0.373 mL hydrogel/well.

### 4.7. Analysis of Cell Viability

To assess the viability of embedded NIH/3T3- and EA.hy926-cells, living cells were stained with Calcein AM and the nuclei were labelled using DAPI staining solution. To prepare the DAPI staining solution, 1 mL of stock solution (1 mg/mL) was diluted 10^3^× using Hank’s Balanced Salt Solution (HBSS). The staining solution for viable cells contained 4 μL Calcein AM per mL HBSS, which was added to the hydrogels and incubated for 45 min at 37 °C in a controlled atmosphere. Fluorescent images were taken using an Axio Observer D1 microscope (Zeiss, Germany) at 10× objective magnification. Lastly, numbers of total and viable cells were calculated using the ImageJ software and automatic cell counting.

### 4.8. Flow-Cytometry Analyses of Cells

Flow cytometry was performed using a Gallios cytofluorometer™ (Beckman Coulter, Fullerton, CA, USA) in order to analyze cell viability, apoptotic and necrotic cell numbers, intracellular reactive oxygen species (ROS) generation and cellular thiol content of primary human cells. The experimental set up and analyses were performed according to the protocol used in a study of Daum et al. [54].

#### 4.8.1. Preparation of 2′,7′-Dichlorofluorescin Diacetate (DCFH-DA) Probe for Intracellular ROS Detection

Detection of intracellular ROS was performed using 2′,7′-Dichlorofluorescin diacetate (DCFH-DA), which is de-esterified intracellularly upon oxidation and turns to highly fluorescent 2′,7′-dichlorofluorescein (DCF). This allows a sensitive and rapid quantification of ROS in response to oxidative metabolism of cells and can be detected at emission wavelength of 523 nm and excitation of 502 nm. All harvested cells were stained with DCFH-DA dye at 20 µM final concentration prior to mixing with hydrogels and were incubated in the dark chamber filled with 5 % CO_2_ at 37 °C for 20 min. After incubation, cells were washed with PBS (10 mL), suspended directly in the respective hydrogel and divided into 24-well plates with a final batch volume of 373 µL. Control cells with and without a DCFH-DA label were grown in parallel on the cell culture plastic surface. After the desired incubation period (6 h, 24 h, 72 h), cells were extracted from cross-linked hydrogels by EDTA treatment for 10 min in the cell culture incubator. After the incubation period, samples were transferred to 15 mL falcon tubes and centrifuged at 300× *g* for 10 min. Supernatant was collected and frozen for FTIR and NMR analysis. Cell pellets were re-suspended in 250 µL PBS, counted using MUSE^®^ Cell Analyzer and transferred into flow cytometer tubes (5 × 10^4^ cells/per sample).

#### 4.8.2. Preparation of Staining Solutions and Analysis of Flow Data

To prepare the staining solution, 5.1 µg/mL DiI, 20 µg/mL PI and 50 μM MBB were dissolved in Ringer’s solution. Cell suspensions containing 5 × 10^4^ cells in 50 µL volume were mixed with 250 µL freshly prepared staining solution and incubated for 30 min at 37 °C.

DiI dye stains the cells that only have intact mitochondrial membrane potential, indicating cell viability, while PI dye labels necrotic cells. Apoptotic cells are determined by gating on the area that is not stained by either DiI or PI dye in the whole cell population. Thus, viable cells were characterized by DiI-positive and PI-negative staining, apoptotic cells were DiI-negative and PI-negative, and necrotic cells were DiI-negative and PI-positive. MBB dye gives a fluorescent signal upon coupling with thiols, including glutathione (GSH), N-acetylcysteine, mercaptopurine, peptides and plasma thiols and the resulting thiol conjugate of monobromobimane has absorption/emission maxima ~394/490 nm. The side scatter value of control cells was set to 100 %, and effects of the tested hydrogels were calculated with reference to that. Mean values of DCF (oxidized form of DCFH-DA) and MBB were calculated by the area that was gated at viable cells. Every sample was measured for a fixed time (40 s, per sample).

Electronic compensation was used to correct for bleed through emission. The data analysis was performed with Kaluza software version 2.0 (Beckman Coulter). All flow cytometry analyses were conducted in three independent experiments, each with triplicate samples. Control cells with DCFH-DA were represented as control data in all graphs.

### 4.9. Immunofluorescence Staining of Samples

Immunofluorescence analysis of Ki-67 in human primary cells was performed to detect proliferating cells. Ki-67 is commonly used as a proliferation marker because it is not detected in G0 cells but increases steadily from G1 through mitosis. The cells were fixed with 4 % paraformaldehyde for 15 min, permeabilized with 0.2% Triton™ X-100 for 5 min and blocked with 1 % FCS for 1 h at room temperature. The cells were labeled with mouse anti-human Ki-67 monoclonal antibody at 1:50 dilution in 1 % FCS, incubated at 4 °C overnight and then stained with goat anti-mouse IgG (H+L) secondary antibody conjugated with Alexa Fluor Plus 488 at a dilution of 1:1000 for 45 min at room temperature. Nuclei were stained with Hoechst 33342 with 5 µg/mL final concentration for 30 min. Samples were washed three times with HBSS, and cells were visualized using fluorescence microscope Zeiss Axio Observer Z1 (Zeiss, Jena, Germany) at 10× magnification.

### 4.10. Statistical Analysis

All experiments were repeated independently three times and run in triplicates. Data obtained from different assays are presented as mean ± standard error of mean (SEM), unless stated otherwise. The analysis of differences between the samples concerning cell viability, apoptotic and necrotic cell determination was performed using one-way ANOVA followed by the Tukey post hoc test for all pairwise multiple comparisons. *p* < 0.05 was considered statistically significant. SigmaPlot^®^ 12.3 Software was used for statistical analyses.

## 5. Conclusions

In this study, we were able to show for the first time that two different cell types in contact with an oxidized alginate hydrogel respond with strikingly different reaction paths. Using alginate oxidized under defined conditions and characterized by FTIR and NMR, we demonstrated that the chemical modifications have an influence on the cellular behavior of fibroblasts and endothelial cells. The free aldehyde groups of ADA trigger pathways of intracellular oxidative stress, which ultimately result in either necrotic cell death in cells with low cellular thiol levels, or recovery and renewed proliferation in cells with a high antioxidant content. The importance of this work for the biofabrication field lies in the controlled bioink synthesis based on ADA-GEL, detailed material characterization and the extensive cell biology investigations. In particular, the interactions between this sensitive system of chemically active groups in ADA-GEL hydrogels and the cellular responses must be considered and precisely adjusted for cell-friendly biofabrication approaches.

## Data Availability

Data supporting reported results will be provided upon request.

## Supplementary Materials

The following are available online at https://www.mdpi.com/1422-0067/22/5/2358/s1.

## Abbreviations

ADA	alginate di-aldehyde
ATR-FTIR	attenuated total reflectance-Fourier transform infrared spectroscopy
DAPI	2-[4-(aminoiminomethyl)phenyl]-1H-indole-6-carboximidamide hydrochloride
DCF	2′,7′-dichlorofluorescein
DCHF-DA	2′,7′-dichlorofluorescein diacetate
DiI	1,1′,3,3,3′,3′-hexamethylindodicarbocyanine iodide
DMEM	Dulbecco’s modified Eagle medium
DNA	deoxyribonucleic acid
DO	degree of oxidation in percent
DPBS	Dulbecco’s phosphate buffered saline
EA.hy926	human endothelial cell line
ECM	extracellular matrix
ECs	endothelial cells
EDTA	ethylenediaminetetraacetic acid
FCS	fetal calf serum
GEL	gelatin
GSH	glutathione
HBSS	Hank’s balanced salt solution
HUVEC	human umbilical vein endothelial cells
MBB	monobromobimane
NIH/3T3	mouse fibroblast cell line
NMR	nuclear magnetic resonance spectroscopy
PI	propidium iodide
ROS	reactive oxygen species
SEM	standard error of mean
