# Supplementary material for: Differential Responses to Bioink-Induced Oxidative Stress in Endothelial Cells and Fibroblasts
Source: Int J Mol Sci. 2021 Feb 26;22(5):2358. doi: 10.3390/ijms22052358 (PMC7956320; doi:10.3390/ijms22052358)
Supplement: Supplementary file 1 [file ijms-22-02358-s001.pdf]

Manuscript ID: ijms-1104071

Differential responses to bioink-induced oxidative stress in endothelial cells and fibroblasts

Hatice Genc, Jonas Hazur, Emine Karakaya, Barbara Dietel, Faina Bider, Jürgen Groll, Christoph Alexiou, Aldo R. Boccaccini, Rainer Detsch, and Iwona Cicha

Supplementary Information

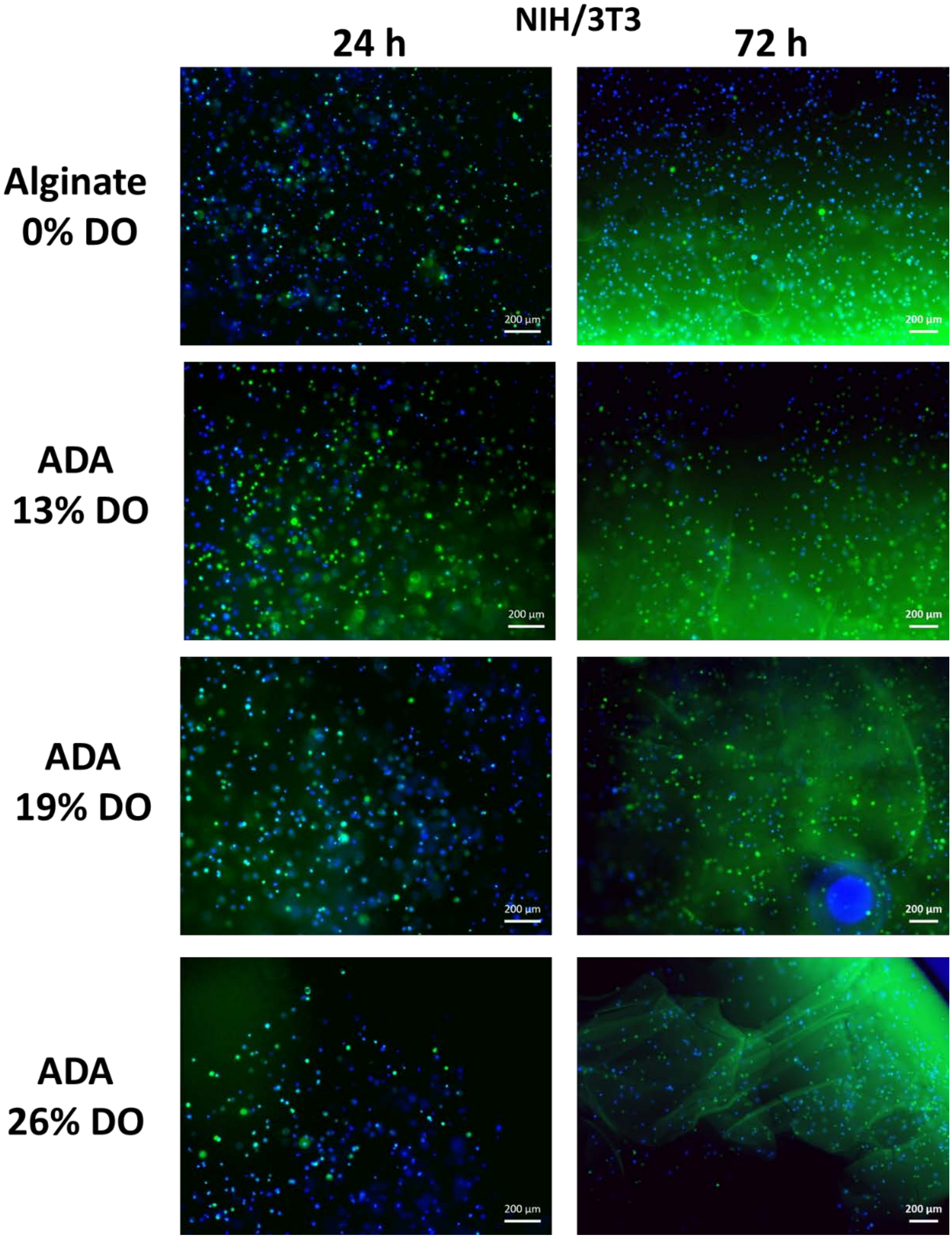

Figure S1: Calcein AM/DAPI staining of NIH/3T3 cells after 24 h or 72 h of incubation in ADA hydrogels with growing degree of oxidation (as indicated). Example images are shown. Nuclei (blue), Calcein (green).

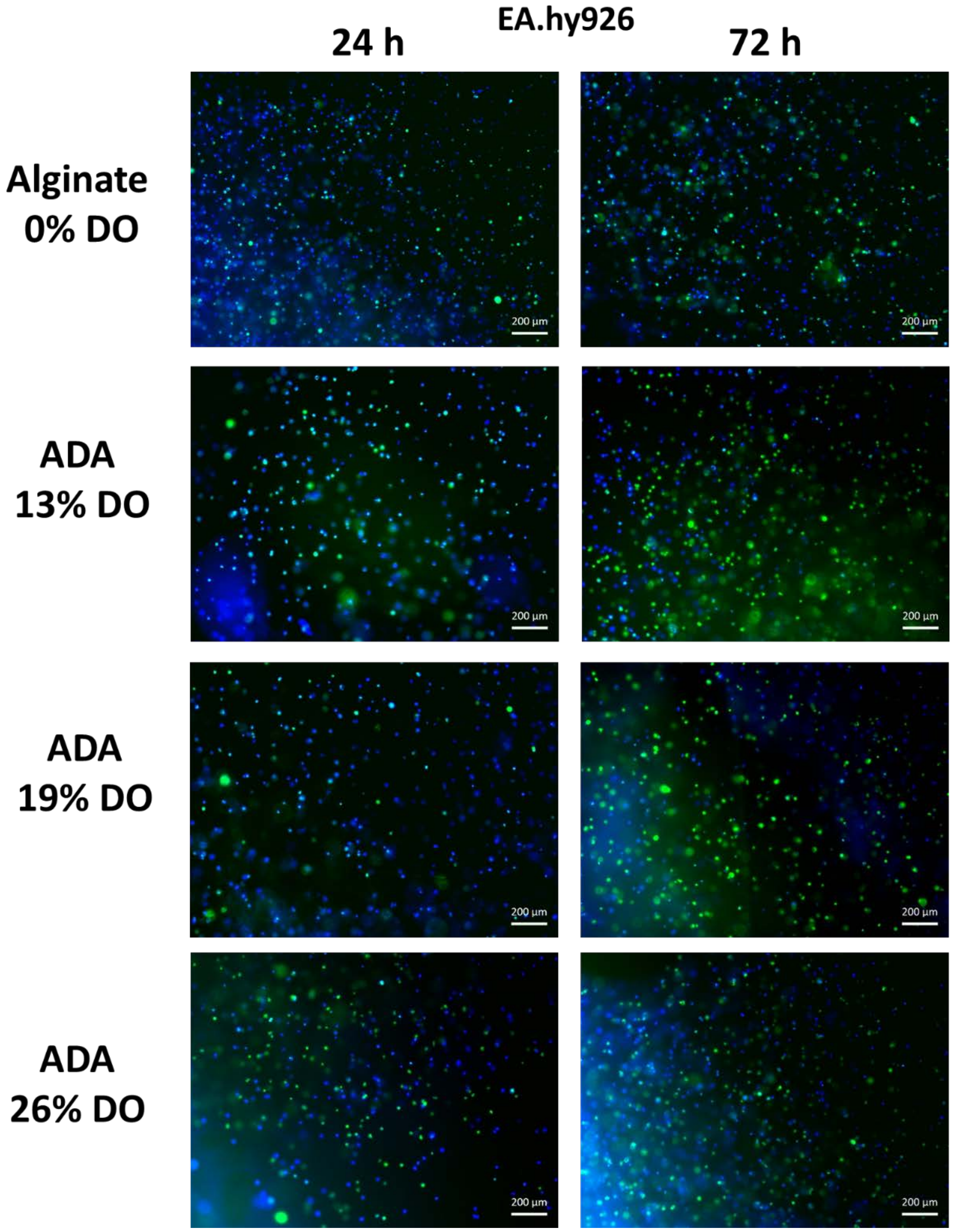

Figure S2: Calcein AM/DAPI staining of Ea.hy926 cells after 24 h or 72 h of incubation in ADA hydrogels with growing degree of oxidation (as indicated). Example images are shown. Nuclei (blue), Calcein (green).
